# CXCR3 as a molecular target in breast cancer metastasis: inhibition of tumor cell migration and promotion of host anti-tumor immunity

**DOI:** 10.18632/oncotarget.6125

**Published:** 2015-10-15

**Authors:** Guiquan Zhu, H. Hannah Yan, Yanli Pang, Jiang Jian, Bhagelu R. Achyut, Xinhua Liang, Jonathan M. Weiss, Robert H. Wiltrout, M. Christine Hollander, Li Yang

**Affiliations:** ^1^ Laboratory of Cancer Biology and Genetics, National Cancer Institute, Bethesda, MD, USA; ^2^ Department of Head and Neck Surgery, Sichuan Cancer Hospital, Sichuan University, Chengdu, P. R. China; ^3^ Center for Reproductive Medicine, Department of Obstetrics and Gynecology, Peking University Third Hospital, Beijing, P. R. China; ^4^ Department of Oral and Maxillofacial Surgery, State Key Laboratory of Oral Diseases, West China Hospital of Stomatology, Sichuan University, Chengdu, P. R. China; ^5^ Cancer and Inflammation Program, National Cancer Institute, Frederick, MD, USA

**Keywords:** CXCR3, tumor metastasis, migration, host immunity, drug treatment

## Abstract

Chemokines and chemokine receptors have critical roles in cancer metastasis and have emerged as one of the targeting options in cancer therapy. However, the treatment efficacy on both tumor and host compartments needs to be carefully evaluated. Here we report that targeting CXCR3 decreased tumor cell migration and at the same time improved host anti-tumor immunity. We observed an increased expression of CXCR3 in metastatic tumor cells compared to those from non-metastatic tumor cells. Knockdown (KD) of CXCR3 in metastatic tumor cells suppressed tumor cell migration and metastasis. Importantly, CXCR3 expression in clinical breast cancer samples correlated with progression and metastasis. For the host compartment, deletion of CXCR3 in all host cells in 4T1 mammary tumor model significantly decreased metastasis. The underlying mechanisms involve a decreased expression of IL-4, IL-10, iNOs, and Arg-1 in myeloid cells and an increased T cell response. IFN-γ neutralization diminished the metastasis inhibition in the CXCR3 knockout (KO) mice bearing 4T1 tumors, suggesting a critical role of host CXCR3 in immune suppression. Consistently, targeting CXCR3 using a small molecular inhibitor (AMG487) significantly suppressed metastasis and improved host anti-tumor immunity. Our findings demonstrate that targeting CXCR3 is effective in both tumor and host compartments, and suggest that CXCR3 inhibition is likely to avoid adverse effects on host cells.

## INTRODUCTION

Metastasis of epithelial tumor cells critically depends on acquisition of a disseminating phenotype that allows tumor cells to migrate, invade, and colonize in distant organs. In addition, the metastatic process also requires the participation of a host compartment [1–4]. Further, systemic suppression of both innate and adoptive immune cells is also paramount in tumor escape from host immune surveillance, which includes compromised function of antigen presenting cells, NK cells, B, and T lymphocytes [5–7]. Despite our recognition of the devastating consequences of metastasis, we have not been able to treat cancer metastasis effectively [8]. One major challenge is the selection of therapy that can not only target cancer cells efficiently, but also avoid an adverse effect on the host compartment and preferably improve host anti-tumor immunity. Our understanding of the molecular mechanisms underlying both tumor and host compartments during the metastatic process is critical for cancer therapy to be more effective and less toxic.

One of the molecular mechanisms involves chemokines/chemokine receptors [9, 10]. The chemokine receptors are a family of 18 to 22 G-protein–coupled receptors whose expressions and functions have been noticed in a number of malignancies [11]. There is clear implication of chemokine receptors in breast cancer metastasis [12, 13]. CXCR3 has been reported to have a metastasis-promoting function in breast cancer [14–16], colon cancer [17–19], and osteosarcoma [20], as well as lung cancer [21]. CXCR3-targeted therapy has been proposed as a treatment option. However, its molecular mechanisms of function and therapeutic application potentials, especially on the host, remain to be investigated. Here we report that CXCR3 KD in tumor cells inhibited tumor cell migration and metastasis. Deletion of CXCR3 in host cells using CXCR3 KO mice showed a decreased metastasis and improved host anti-tumor immunity. Treating tumor-bearing mice with a CXCR3 inhibitor (AMG487) that targets both tumor and host compartments decreased tumor metastasis and simultaneously improved host immune responses. Our study, using both genetic and chemical approaches, demonstrates that CXCR3 inhibition could inhibit tumor cell metastatic capability and, at the same time, improve host anti-tumor immunity. Our data suggest that CXCR3 inhibition, unlike most chemotherapy agents, should prevent adverse or toxic effect on host cells.

## RESULTS

### CXCR3 expression was increased in the metastatic mammary tumor cells

**Figure 1:**
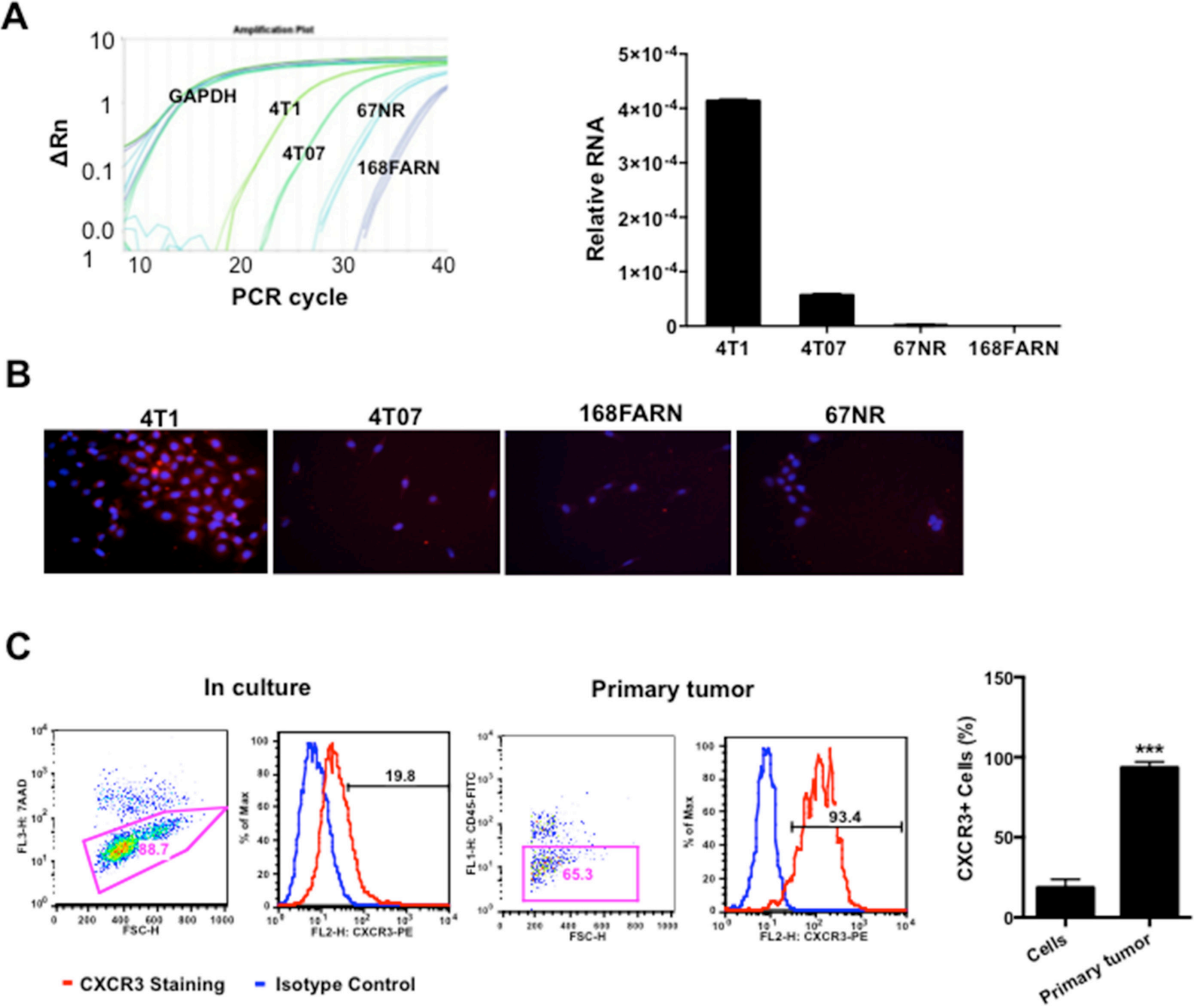
CXCR3 is increased in metastatic mammary tumor cells **A.** Relative expression of CXCR3 in metastatic 4T1 cells and low metastatic derivatives, by Q-PCR; triplicate per sample. **B.** Representative microscopy of CXCR3 immunofluorescence staining in 4T1 cells and relatively low or non-metastatic derivatives cultured in chamber slides. **C.** Flow cytometry analysis of CXCR3 expression in single cell suspension from cultured 4T1 cells and 4T1 primary tumor tissues. Shown is one of the three experiments performed. ***P < 0.001.

CXCR3 has been reported to play a role in tumor progression and metastasis in a number of cancers [21]. To investigate the role of CXCR3 in breast cancer metastasis, we used the 4T1 mammary tumor model, which shares many characteristics with human breast cancer, particularly its ability to spontaneously metastasize to the lungs. The 4T1 model also has three additional cell lines derived from the same tumor but with different degrees of malignancy— 4T1 being the most malignant, then 4T07, 168FARN, and 67NR (the least malignant). To examine whether there is correlation between CXCR3 expression and metastatic ability, we first performed Q-PCR to compare the expression of CXCR3 in these cell lines. Interestingly, CXCR3 expression was the highest in 4T1 cells, second highest in 4T07, then much lower in 168 FARN or 67NR (Figure 1A). The results were further validated with immunofluorescence staining of CXCR3 in cultured 4T1, 4T07, 168FARN, and 67NR cells (Figure 1B). These data suggest a correlation of CXCR3 level with the degree of the malignancy in cultured cell lines.

It is well known that tumor microenvironment is an indispensible participant in tumor metastasis. To examine whether the tumor microenvironment has an effect on CXCR3 expression, we next examined CXCR3 expression level in tumor cells from primary tumor tissues compared to those in culture. The tumor cells derived from the tumor tissues had significantly higher expression of CXCR3 compared the tumor cells in culture when the same non-enzymatic dissociation procedure was applied to the preparation of the single cell suspension for flow cytometry analysis (Figure 1C). This observation was also made in the B16F10 melanoma mouse model (Supplementary Figure 1). Together these data suggest that CXCR3 is likely important in tumor metastasis and its expression is likely up-regulated by the tumor microenvironment.

### CXCR3 knockdown in tumor cells significantly decreased 4T1 metastasis as well as tumor cell migration and mobility

**Figure 2:**
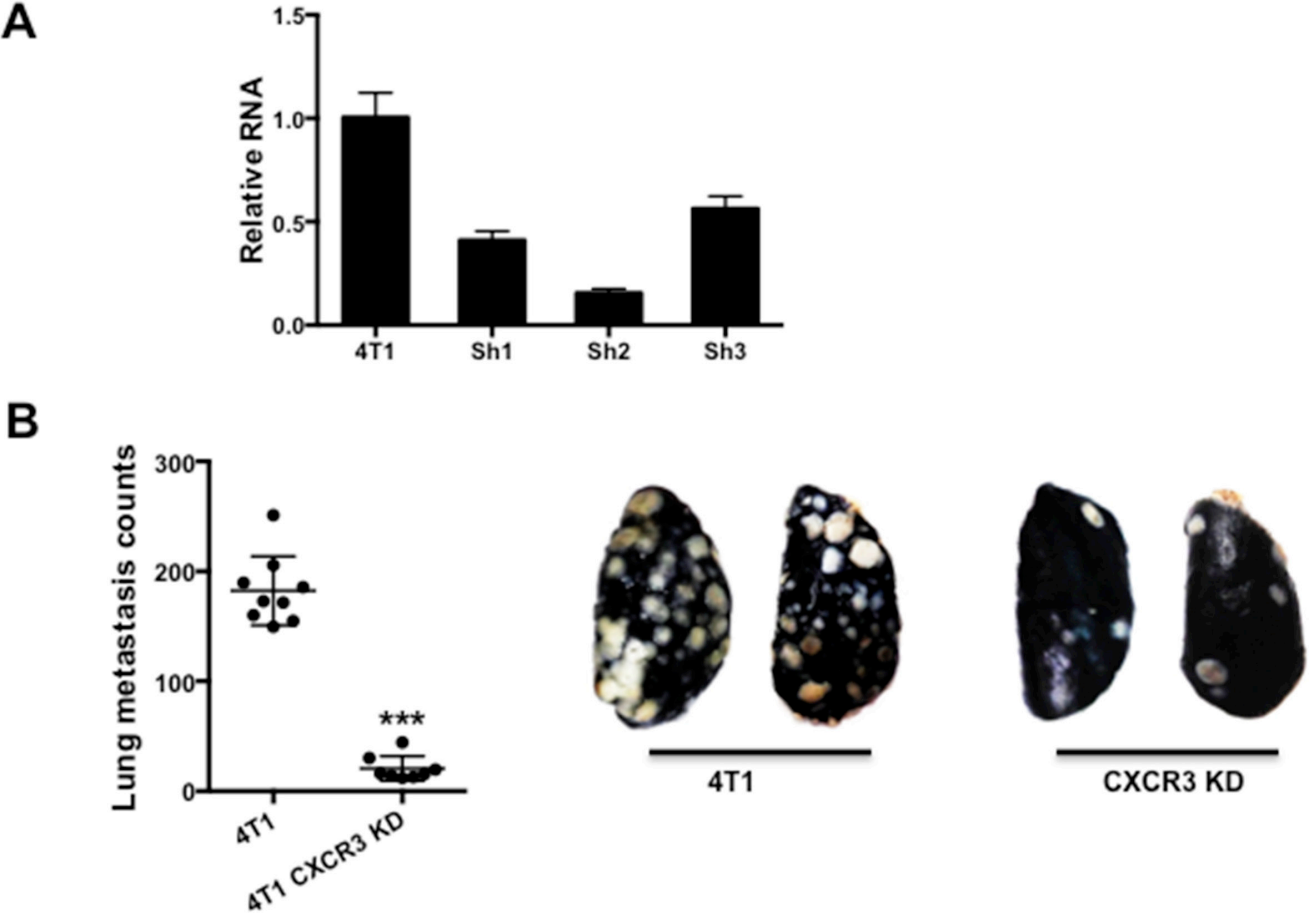
CXCR3 promotes metastasis **A.** Q-PCR of CXCR3 in 4T1 tumor cells with or without shRNA KD. **B.** Left panel: Number of lung metastasis nodules from mice that received tail vein injection of 4T1 or CXCR3 KD 4T1 cells; 8–9 mice per group. Data are represented as mean +/− SEM. Right panel: Representative pictures of lung metastasis nodules. White dots indicate lung metastasis. *P < 0.05.

To understand the function of CXCR3 in breast cancer metastasis, we knocked down (KD) CXCR3 in 4T1 cells using shRNA (Figure 2A). We then injected these cells into the tail vein of syngeneic Balb/c mice. Mice bearing 4T1 CXCR3 KD cells had significantly reduced lung metastasis compared to the controls (Figure 2B), suggesting CXCR3 plays a critical role in promoting tumor metastasis.

**Figure 3:**
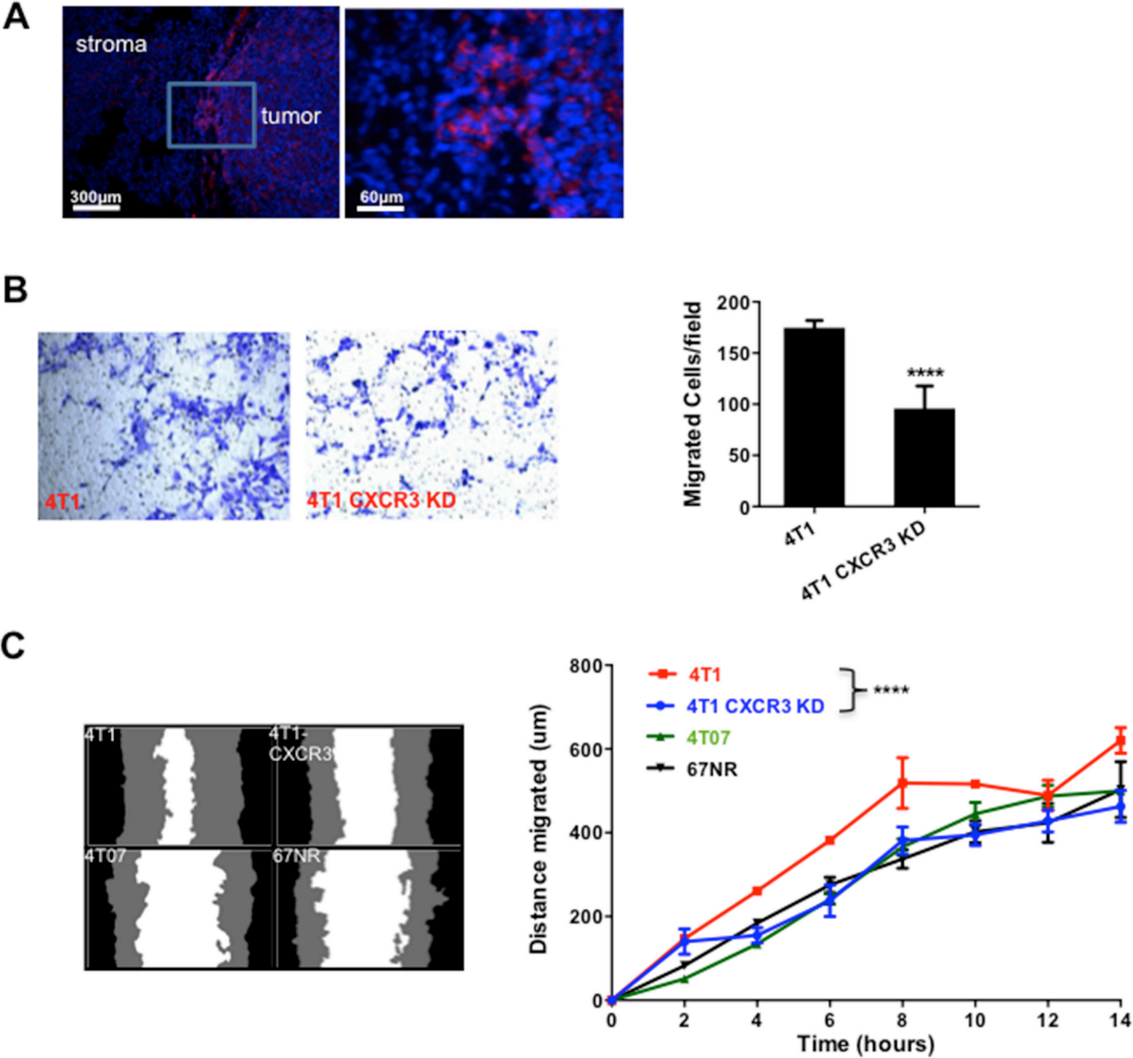
CXCR3 KD inhibited tumor cell migration **A.** CXCR3 immunofluorescence staining of tumor sections at invasive front of 4T1 tumor tissues. **B.** Transwell migration assay of control and CXCR3 KD 4T1 cells. The cells on the underside of the filter (images on the left panels) were counted and plotted in the bar figure (right panel). **C.** Scratch assay of 4T1 and CXCR3 KD 4T1 cells, as well as 4T07 and 67NR cells. Representative images of wound closure are on the left. The distance migrated in 14 h is plotted on the right. The distance migrated was calculated as Wo-Wt, where Wo represents the original width of the wound and Wt is the width of the wound at each time point. Shown is one of two experiments performed. Data are represented as mean +/− SEM. ****P < 0.0001.

We next investigated the mechanisms that are responsible for CXCR3's role in metastasis. Using immunofluorescence staining of the tumor tissues, we initially observed a higher level of CXCR3 at the invading edge of the tumors (Figure 3A). This observation led us to hypothesize that CXCR3 promotes tumor cell invasion and migration, critical steps in the metastatic cascade. We thus performed a Transwell migration assay in which the 4T1 tumor cells, with or without CXCR3 KD, were tested for their migratory capability. CXCR3 KD diminished the 4T1 cell migration (Figure 3B). To further examine this, we conducted a scratch or wound healing *in vitro* assay using IncuCyte (Essen BioScience, Ann Arbor, MI), which allowed us to monitor cell migration and wound closure in real time by taking a series of pictures over a specified period. As expected, the 4T1 cells showed better migration and wound closure than the non-metastatic 4T07 and 67NR cells (Figure 3C). Interestingly and consistently, CXCR3 KD decreased the migration and wound closure of 4T1 cells (Figure 3C, left panel for representative figures and right panel for time course studies). CXCR3 KD also changed the morphology of 4T1 cells in culture (Supplementary Figure 2). Together, these data suggest that CXCR3-mediated signals likely promote tumor cell migration and mobility, and contribute to metastasis.

### Correlation of CXCR3 with human breast cancer progression and metastasis

**Figure 4:**
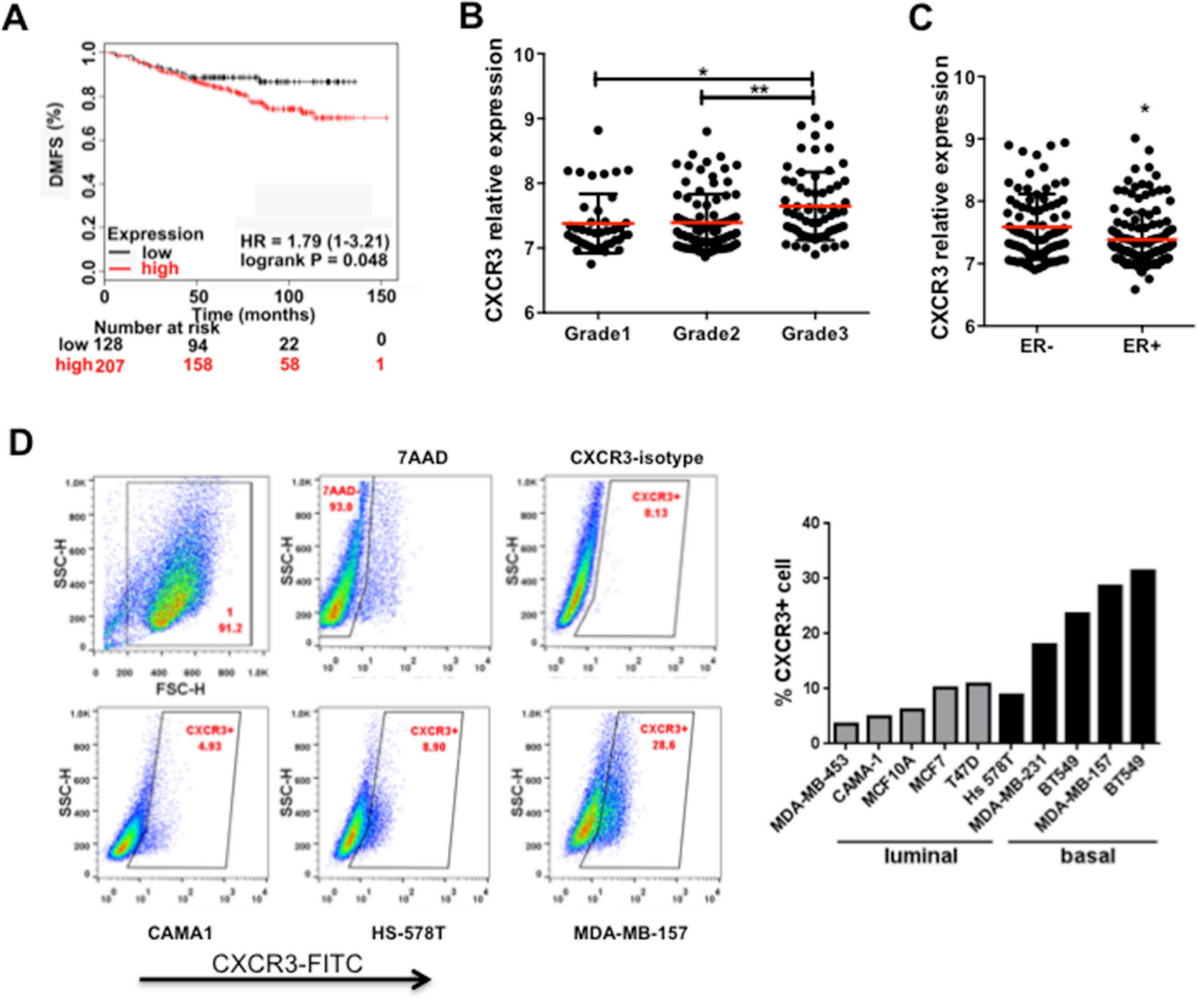
CXCR3 expression correlates with breast cancer progression and metastasis **A.** Kaplan-Meier survival curve for the correlation of CXCR3 expression level with distant metastasis-free survival of breast cancer patients (GEO database) [22]. The high or low CXCR3 expression was defined as above or below the average of CXCR3 expression in all patients. **B.** CXCR3 expression in breast cancer patients with different tumor grades (GSE22220). **C.** CXCR3 expression in ER – and ER + breast cancer patients (GSE22220). Breast cancer data sets were analyzed by GeneSpring GX 10.0 software. Data are represented as mean +/− SEM. *P < 0.05; **P < 0.01. **D.** Flow cytometry analysis of CXCR3 expression in a panel of human breast cancer cell lines. Representative samples are on the left; all data are in the bar figure on the right.

To understand the clinical relevance of our mouse studies, we investigated the possible correlation between CXCR3 expression with human breast cancer progression. First we utilized Kaplan-Meier Plotter to evaluate the prognostic utility of 22,277 genes in 1,809 breast cancer patients [22]. The higher CXCR3 expression level correlated with a poor distant metastasis free survival (DMFS) of patients with ER + tumors treated by Tamoxifen (Figure 4A). Next we examined the correlation of CXCR3 with other clinical-pathological characteristics in the publicly available database GSE22220 [23] using GeneSpring GX 10.0 software. We used the average of CXCR3 expression in all patients as a cut-off; the results above the average were categorized as the CXCR3 high group whereas the results below the average were categorized as the CXCR3 low group. CXCR3 level correlated with tumor grades (Figure 4B). Grade 3 tumors showed significantly higher CXCR3 level than grade 1 or grade 2 (Figure 4B). Furthermore, CXCR3 was differentially expressed in ER- and ER + breast cancer patients (GSE22220). ER- patients, who often have a worse prognosis than that of the ER + patients, showed a significantly higher level of CXCR3 than ER + patients (Figure 4C). The CXCR3 expression level was clearly higher in the basal cancer types than in the luminal types in 10 human breast cancer cell lines examined using flow cytometry analysis (Figure 4D). These data independently confirm that increased CXCR3 expression correlates with breast cancer progression in a clinical setting, and indicate that anti-CXCR3 treatment could provide options for metastasis treatment of breast cancer.

### Host CXCR3 promotes 4T1 lung metastasis and immune suppression

**Figure 5:**
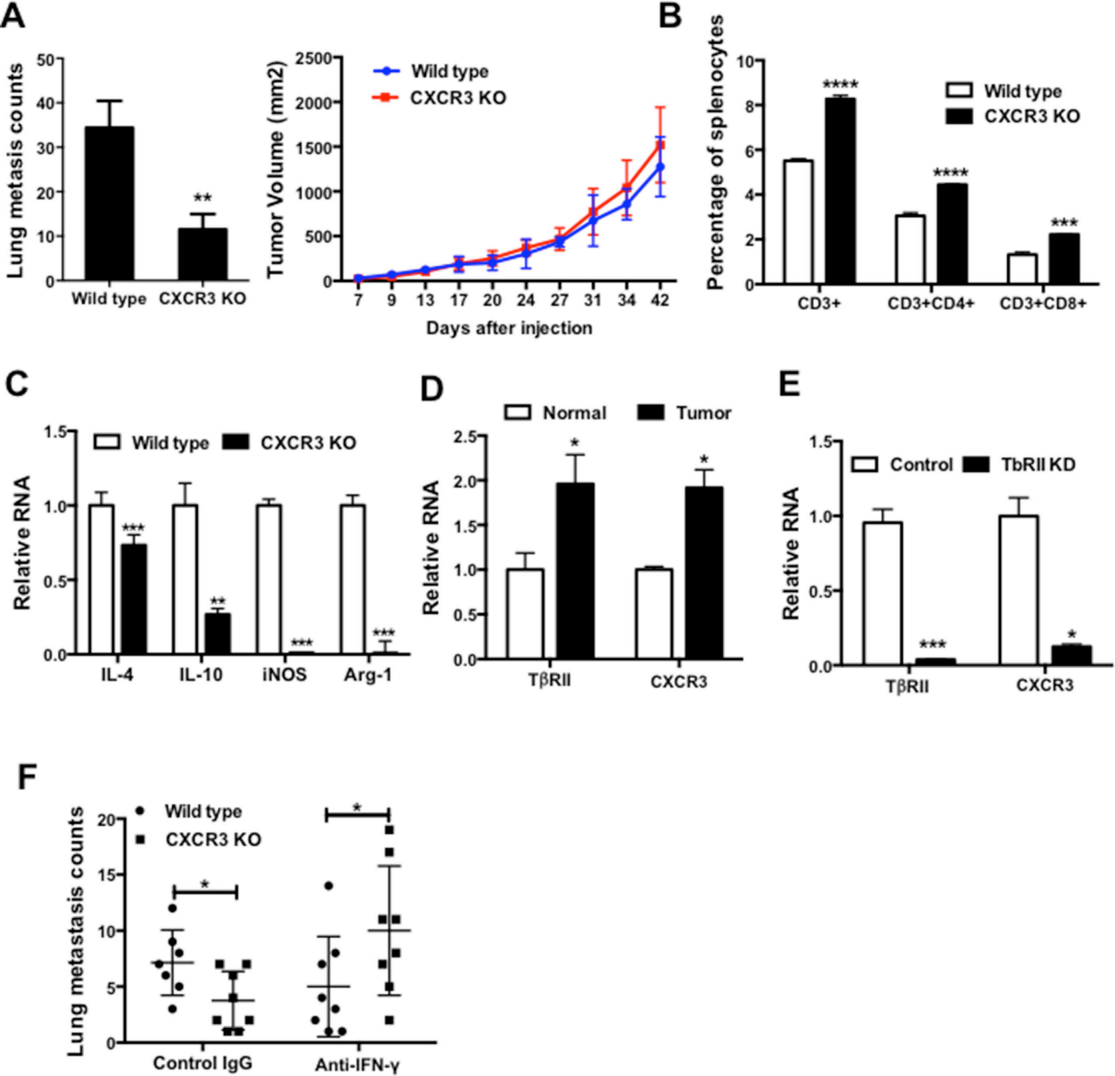
CXCR3 promotes lung metastasis and impairs host anti-tumor immunity **A.** Lung metastasis counts (left panel) and primary tumor growth (right panel) of wild type and CXCR3 KO mice received 4T1 cell injection in mammary fat pad; 5–6 mice per group. **B.** Percentage of T cells and subsets in splenocytes of wild type and CXCR3 KO mice; 3–4 mice per group. **C.** The expressions of IL-4 and IL-10, as well as Arg1 and iNOS, in myeloid cells from wild type and CXCR3 KO mice; 3–4 mice per group. **D.** Q-PCR of TbRII and CXCR3 in myeloid cells from normal and tumor-bearing mice; 3–4 mice per group. **E.** Q-PCR of TbRII and CXCR3 in RAW264.7 macrophages with or without TbRII KD; triplicate per sample. **F.** Lung metastasis counts of wild type and CXCR3 KO mice bearing 4T1 tumors, with IFN-γ neutralization or IgG controls; 7–8 mice per group. Data are represented as mean +/− SEM. *P < 0.05; **P < 0.01; ***P < 0.001; ****P < 0.0001.

Many therapeutic drugs developed to target cancer cells often show adverse effects on host cells. To examine the possible effect of anti-CXCR3 treatment on the host compartment, we first examined CXCR3 KO mice in which the CXCR3 receptor is deleted in all host cells, which models the pan effect of CXCR3 chemical inhibitor on all host cells. Deletion of CXCR3 decreased the number of lung metastases in mice that received 4T1 tumor injection in #2 mammary fat pad (Figure 5A, left panel), with no effect on the primary tumor size (Figure 5A, right panel). Further, CXCR3 was expressed in most of the host immune cells including Gr-1 + CD11b + immature myeloid cells, F4/80 macrophages, B cells, CD4, and CD8 T cells (Supplementary Figure 3A). These data indicate that signals mediated through CXCR3 may affect host immune responses. Indeed, the percentages of the CD3 +, CD3 + CD4 +, and CD3 + CD8 + cells in the spleen of CXCR3 KO mice were significantly higher than those in the wild type control mice (Figure 5B). Additionally, myeloid cells sorted from CXCR3 KO mice showed reduced expressions of IL4 and IL10, as well as iNOS and arginase1 (Figure 5C). These data suggest that CXCR3 deletion could direct the myeloid cells into more of a type 1 phenotype, thus stimulating host anti-tumor immunity. Interestingly, we observed a correlation of CXCR3 level with TβRII expression (Figure 5D) in myeloid cells that we previously reported play a critical role in breast cancer metastasis and host immune suppression [24]. KD of TβRII in RAW264.7 macrophages significantly decreased CXCR3 (Figure 5E), suggesting a regulatory role of TGF-β in CXCR3 expression. Finally, IFN-γ neutralization diminished the metastasis inhibition in the CXCR3 KO mice bearing 4T1 tumors, which was not seen in the wild type mice (Figure 5F). The differential effect of IFN-γ neutralization between CXCR3 KO and wild type was not observed in the primary tumor growth (Supplementary Figure 3B). These data suggest that CXCR3 mediated signaling suppressed IFN-γ production and T cell expansion, thus contribute to lung metastasis.

### CXCR3 specific inhibitor AMG487 attenuates 4T1 lung metastasis through effect on both tumor and host compartment

**Figure 6:**
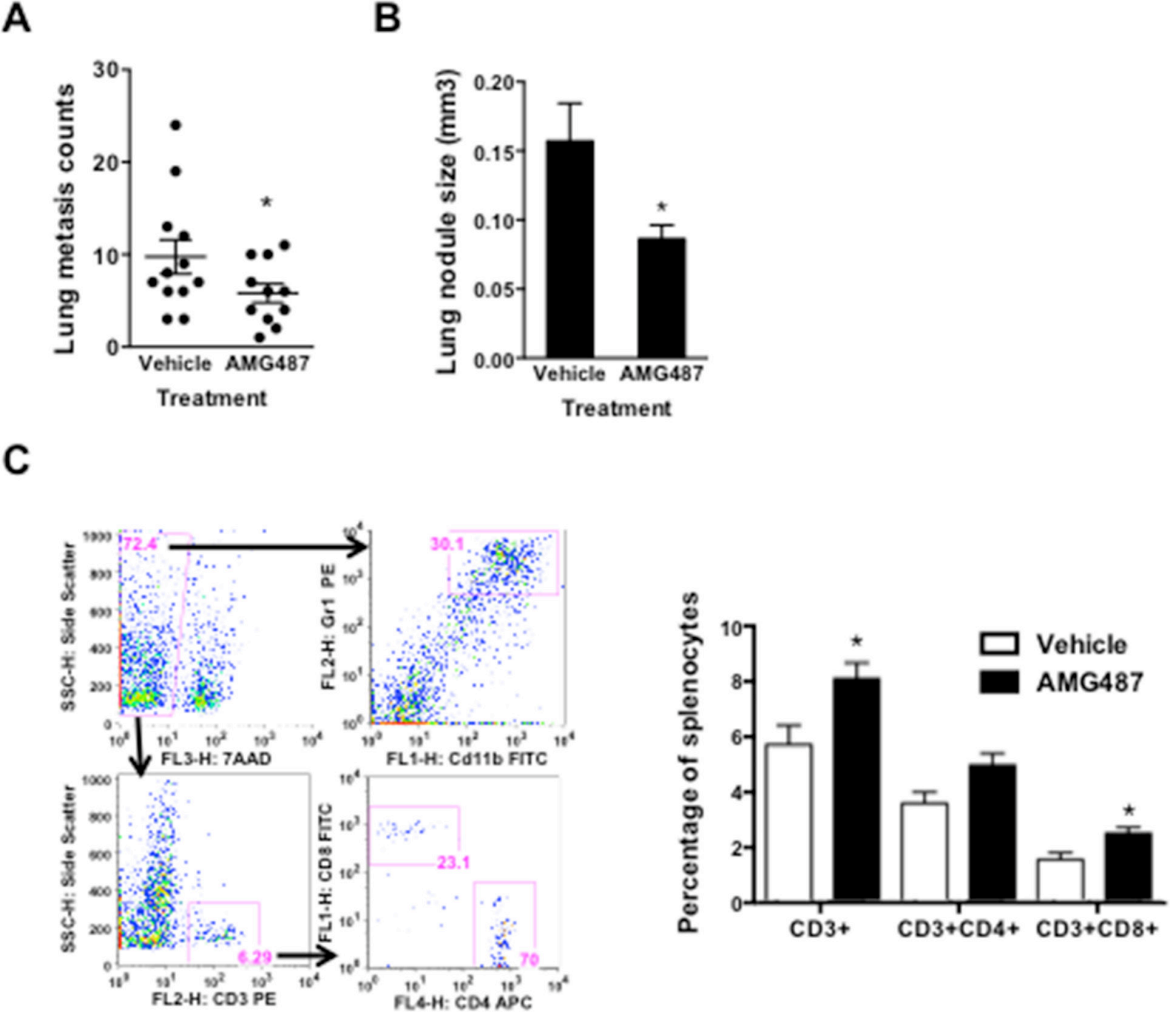
The anti-tumor effect of CXCR3 inhibitor AMG487 on both tumor and host compartments **A-B.** Lung metastasis nodule counts A. and the size of metastatic nodules **B.** of tumor-bearing mice treated with vehicle or AMG487; 11–12 mice per group. **C.** Flow cytometry analysis of T cells and subsets in 4T1 tumor-bearing mice treated with AMG487 or vehicle control; 6 mice per group. Data are represented as mean +/− SEM. *P < 0.05.

AMG487 is a specific small molecular inhibitor of CXCR3, and it has significant inhibitory effect on tumor progression including breast cancer [14], colon cancer [18], and osteosarcoma [20]. However, the effect of AMG487 on both tumor and host compartment needs to be carefully evaluated. This is because cancer therapies targeting metastasis not only target tumor cells but also unavoidably target the host compartment. Here we focused on the effect of AMG487 on the immune system of the tumor-bearing host. Mice bearing 4T1 tumors were injected with AMG487 intraperitoneally (5 mg/kg/dose) twice daily. The tumor growth, lung metastasis, and immune cell responses were evaluated after 28 days. AMG487 decreased the number of lung metastasis (Figure 6A) and the size of metastasis nodules (Figure 6B), but did not have an effect on primary tumor growth (Supplementary Figure 4). Mice treated with AMG487 showed increased CD3 + CD4 + and CD3 + CD8 + cell numbers in the peripheral blood (Figure 6C), with no change in the number of Gr-1 + CD11b + myeloid cells (data not shown). These data suggest that AMG487 not only targeted cancer cells directly as reported [14] but also improved host immune responses, thus alleviating the adverse effect on host immunity often seen in small molecular inhibitor-based cancer treatment.

## DISCUSSION

Therapeutic treatments of cancers not only target tumor cells but also unavoidably affect the host compartment. CXCR3 inhibition has emerged as one of the metastasis-targeting options. Here we evaluate the effect of CXCR3 targeting on both the tumor cells and the host compartments. We report that genetic targeting of CXCR3 in both tumor cells and host-derived cells showed tumor-inhibitory effect. CXCR3 targeting with the small molecular inhibitor AMG487 significantly reduced metastasis and improved host anti-tumor immunity. The underlying mechanisms involved decreased tumor cell migration and mobility, and improved myeloid cell function and T cell response. Our data provide molecular insight for CXCR3 targeting in metastasis disease treatment. In addition, our work demonstrates that CXCR3 inhibition may provide double benefits for inhibiting tumor and improving host immunity, unlike most agents that are effective in targeting tumor cells but are toxic to host cells.

The increased expression of CXCR3 has been correlated with poor prognosis in breast, melanoma, colon, and renal cancer patients [25]. It has been reported that of the three variants of CXCR3—CXCR3A, CXCR3B, and CXCR3-alt, the two primary variants—CXCR3A and CXCR3B [26]—induce opposite physiological functions [25, 27]. CXCR3A appears to mediate pro-tumor effect including cell proliferation, survival, chemotaxis, invasion, and metastasis; whereas CXCR3B mediates anti-tumor effect via promoting growth suppression, apoptosis, and vascular involution [25]. Notably, one recent study reported that CXCR3B likely promotes stem function; whereas CXCR3A shows pro-proliferative and metastasis-promoting functions [28]. Here in our study of mouse models of breast tumor metastasis, targeting mouse CXCR3, the CXCR3A form, decreased tumor metastasis (Figure 2). Importantly, CXCR3 expression correlates with human breast cancer progression and metastasis (Figure 4). Our finding is in agreement with CXCR3 metastasis-promoting function in breast cancer [14–16], colon cancer [17–19], and osteosarcoma [20], as well as lung cancer [21]. In breast cancers, the molecular mechanisms of CXCR3-mediated metastasis involve tumor-host interaction; for example, mesenchymal stem cells (MSC) were recruited to the tumor microenvironment through CXCL10/CXCR3 axis (MSC/tumor cell), one of the critical signaling loops mediated by hypoxia-inducible factors and important in stromal and tumor cell interaction [29, 30]. CXCL10 facilitates trafficking of CXCR3-expressing cancer cells to bone, and promotes osteolytic bone metastasis [31], implying host involvement. These studies and the effect of CXCR3 inhibition that unavoidably targets the host compartment led us to look into the contribution of host immune response using CXCR3 KO mice under tumor conditions.

The effect of CXCR3 on the host immune system has been recognized as one of the earliest studies showing that CXCR3 KO mice had profound resistance to development of acute allograft rejection [32]. In our study, using genetic approaches of RNA interference and KO mice, as well as a small molecular inhibitor, we found that targeting CXCR3 not only directly inhibited tumor cell migration and mobility, but also improved host immune responses. Our data support that CXCR3 deletion promoted type 1 myeloid cell polarization producing less immune suppressive factors, which in turn enhanced host immune responses (Figure 5). This is consistent with published reports in which the effect of CXCR3 small molecular inhibitor AMG487 depends on Natural Killer cells as NK depletion compromised AMG487 anti-metastatic activity [14]. This is also in agreement with reported enhanced Natural Killer cell function [16]. However, in disagreement with these publications, and our data, CXCR3-dependent anti-tumor response has also been reported. The underlying mechanisms seemed to involve the CXCR3-mediated signaling for migration and infiltration of the activated T cells [33–36]. We anticipate that this pro- or anti-tumor metastasis function is likely context dependent, which is influenced by the cues of multiple CXCL chemokines in the tumor microenvironment. For example, CXCR3 was shown to have an angiostatic effect through CXCL4- or PF4-mediated signaling [14, 37, 38]. Nevertheless, our data demonstrate that the totality or net effect of targeting CXCR3 seemed to inhibit 4T1 metastasis and improve host immune response.

## MATERIALS AND METHODS

### Cell lines and mice

Murine 67NR, 168FARN, 4TO7, 4T1, and B16F10, as well as human breast cancer cell lines, were obtained from ATCC (American Type Culture Collection, Manassas, VA) and kept in liquid nitrogen when not in use. Cells were thawed, cultured, and passaged less than six months for experiments. Balb/c CXCR3 KO mice were originally provided by Dr. Craig Gerard [39]. Female Balb/c or C57BL/6J mice that were 6–8 weeks old, 16–20 g body weight, were obtained from Charles River (Germantown, MD). All animal studies were approved by the National Cancer Institute Animal Care and Use Committee.

### Flow cytometry and cell sorting

For CXCR3 flow cytometry analysis, single cell suspensions were made from primary tumor tissues as described [40], labeled with CXCR3 antibody or isotype control (R&D System, FAB1685P or IC006P), and analyzed on a FACS Calibur flow cytometer (BD, San Jose, CA). The single cell suspensions from cultured human breast cancer cell lines, 4T1 cells, or tumor cells from tumor tissues were isolated by incubating with 0.2 g/L EDTA (Life Technologies, Carlsbad, CA), a non-enzymatic approach, at 37°C for about 15 min. The tumor cells were gated on 7AAD negative (exclusion of dead cells), CD45 negative (exclusion of immune cells), and high SSC and FSC scatter (largely tumor cells). For immune cells, single cell suspension was made from spleens of tumor-bearing CXCR3 KO mice or AMG487 treated mice, and labeled with CD3, CD4, CD8, Gr-1, and CD11b antibodies, followed by flow cytometry analysis or sorting by FACSAria flow cytometer (BD, San Jose, CA) or MACS (Magnetic-activated cell sorting, Miltenyi Biotec, San Diego, CA).

### Immunofluorescence (IF) staining

The tumor cells were cultured on chamber slides, which were incubated with rat anti-CXCR3 antibodies (Santa Cruz Biotechnology, Y-16, 1:100 dilution) followed by Alexa fluor 488 goat anti-rat or Alexa Fluor^®^ 594 donkey anti goat IgG (1:200, Invitrogen) for 1 h.

### Quantitative RT-PCR

Total RNA was extracted from tumor cell lines and sorted Gr-1 + CD11b + cells using an RNeasy Mini Kit (Qiagen). cDNA was synthesized using SuperScript^tm^ First-Strand Synthesis System (Invitrogen). Relative gene expression was determined using iCycler-iQ SYBR Green PCR kit (Bio-Rad). Primer sequences are available upon request.

### shRNA knockdown of CXCR3

Three different lentiviral shRNA constructs were purchased from Open Biosystems. These vectors were packaged into lentivirus with the packaging vectors, pMD2, pRSV-REV, and pMDLg in HEK293T cells. Lentivirus in the supernatant of HEK293T cells were harvested and stored at −80°C. Tumor cells (4T1) were infected using the lentivirus mixture, and selected with puromycin (4 μg/ml, Invitrogen). The KD efficiency of CXCR3 in the stable infected cells was evaluated by q-PCR.

### Scratch and transwell migration assays

For Scratch assay, the tumor cells were seeded on 96-well plates (3 × 10^4^/well) in DMEM with 10% FBS. Eight hours later, cells were starved in serum-free condition overnight. On the second day, the wound was created in a straight line using the 96-well wound maker (IncuCyte, Essen BioScience, Ann Arbor, MI). The width of the wound was monitored by IncuCyte Zoom (Essen BioScience, Ann Arbor, MI) and images were taken every 3 h. The distance migrated was calculated as Wo-Wt, where Wo represents the original width of the wound and Wt is the width of wound at each time point. For migration assay, tumor cells (5 × 10^4^ cells per well) were seeded on the chamber of a 24-well transwell insert (8 μM, Corning) in DMEM containing 2% FBS. The plates were incubated for 6 h at 37°C with 5% CO_2_. Migrated tumor cells were fixed by formalin for 10 min and stained using 0.1% Crystal Violet. The migrated cells, in 4 random fields under a 10 × objective lens, were counted and the average cell number was calculated.

### Spontaneous and experimental metastasis

For the orthotopic model of metastasis, 4T1 cells (5 × 10^4^) were injected into the #2 MFP of Balb/c female mice. Mice were sacrificed 42 days later for evaluation of metastasis and tumor growth. For B16F10 orthotopic model, 1 × 10^6^ B16F10 cells were injected subcutaneously, tumors were removed at day 16, and the mice were euthanized at day 22. Tumor size was measured at 3–4 day intervals using calipers as: Volume = length × width^2^ × 0.5. For experimental metastasis, mice received tail vein injection (TVI) of 4T1 or 4T1 CXCR3 KD cells (2 × 10^5^). The number of lung metastasis was evaluated by whole lung mounting [41] or India ink staining [42] when mice died, became moribund, or when the primary tumors reached a size of 2.0 cm in diameter. For IFN-γ neutralization, the mice were injected intraperitoneally with IFN-γ neutralizing antibody XMG-6 or IgG control, 1 mg per mouse on day 1, 3, and 6 and 0.5 mg on day 9, 12, 15, 18, 21, 24, and 27. Mice were sacrificed on day 28, and tumor growth, as well as lung metastases, were evaluated. For AMG487 treatment, Mice injected with 4T1 tumor cells in mammary fat pad #2 or #4 were injected with AMG487 intraperitoneally (5 mg/kg/dose) twice a day on day 13–17, and once daily thereafter. AMG487 was prepared in 20% hydroxypropyl- b-cyclodextrin in water. The tumor growth, lung metastasis, and immune cell responses were evaluated on day 28.

### Human correlative studies

Human breast cancer databases GEO [22] and GSE22220 were used to investigate the correlation of CXCR3 expression with breast cancer patient survival, tumor grades, or ER- and ER + status. The data sets were analyzed by GeneSpring GX 10.0 software.

### Statistical analysis

Graphpad Prism v5.04 was used for the graphs and for statistics. All data, other than indicated, were analyzed using the Student's *t*-test or one-way ANOVA, and were expressed as mean ± SE. Differences were considered statistically significant when the *p*-value was < 0.05.

## SUPPLEMENTARY MATERIAL FIGURES



## References

[1] Balkwill F, Coussens LM (2004). Cancer: an inflammatory link. Nature.

[2] McAllister SS, Weinberg RA (2010). Tumor-host interactions: a far-reaching relationship. J Clin Oncol.

[3] Cook LM, Hurst DR, Welch DR (2011). Metastasis suppressors and the tumor microenvironment. Semin Cancer Biol.

[4] Kang Y, Pantel K (2013). Tumor cell dissemination: emerging biological insights from animal models and cancer patients. Cancer Cell.

[5] Swann JB, Smyth MJ (2007). Immune surveillance of tumors. J Clin Invest.

[6] Gabrilovich DI, Nagaraj S (2009). Myeloid-derived suppressor cells as regulators of the immune system. Nat Rev Immunol.

[7] Grivennikov SI, Greten FR, Karin M (2010). Immunity, inflammation, and cancer. Cell.

[8] Sethi N, Kang Y (2011). Unravelling the complexity of metastasis - molecular understanding and targeted therapies. Nat Rev Cancer.

[9] Balkwill F (2004). Cancer and the chemokine network. Nat Rev Cancer.

[10] Raman D, Sobolik-Delmaire T, Richmond A (2011). Chemokines in health and disease. Exp Cell Res.

[11] Sarvaiya PJ, Guo D, Ulasov I, Gabikian P, Lesniak MS (2013). Chemokines in tumor progression and metastasis. Oncotarget.

[12] Muller A, Homey B, Soto H, Ge N, Catron D, Buchanan ME, McClanahan T, Murphy E, Yuan W, Wagner SN, Barrera JL, Mohar A, Verastegui E (2001). Involvement of chemokine receptors in breast cancer metastasis. Nature.

[13] Ali S, Lazennec G (2007). Chemokines: novel targets for breast cancer metastasis. Cancer Metastasis Rev.

[14] Walser TC, Rifat S, Ma X, Kundu N, Ward C, Goloubeva O, Johnson MG, Medina JC, Collins TL, Fulton AM (2006). Antagonism of CXCR3 inhibits lung metastasis in a murine model of metastatic breast cancer. Cancer Res.

[15] Walser TC, Ma X, Kundu N, Dorsey R, Goloubeva O, Fulton AM (2007). Immune-mediated modulation of breast cancer growth and metastasis by the chemokine Mig (CXCL9) in a murine model. J Immunother.

[16] Ma X, Norsworthy K, Kundu N, Rodgers WH, Gimotty PA, Goloubeva O, Lipsky M, Li Y, Holt D, Fulton A (2009). CXCR3 expression is associated with poor survival in breast cancer and promotes metastasis in a murine model. Mol Cancer Ther.

[17] Kawada K, Hosogi H, Sonoshita M, Sakashita H, Manabe T, Shimahara Y, Sakai Y, Takabayashi A, Oshima M, Taketo MM (2007). Chemokine receptor CXCR3 promotes colon cancer metastasis to lymph nodes. Oncogene.

[18] Cambien B, Karimdjee BF, Richard-Fiardo P, Bziouech H, Barthel R, Millet MA, Martini V, Birnbaum D, Scoazec JY, Abello J, Al Saati T, Johnson MG, Sullivan TJ (2009). Organ-specific inhibition of metastatic colon carcinoma by CXCR3 antagonism. Br J Cancer.

[19] Murakami T, Kawada K, Iwamoto M, Akagami M, Hida K, Nakanishi Y, Kanda K, Kawada M, Seno H, Taketo MM, Sakai Y (2013). The role of CXCR3 and CXCR4 in colorectal cancer metastasis. Int J Cancer.

[20] Pradelli E, Karimdjee-Soilihi B, Michiels JF, Ricci JE, Millet MA, Vandenbos F, Sullivan TJ, Collins TL, Johnson MG, Medina JC, Kleinerman ES, Schmid-Alliana A, Schmid-Antomarchi H (2009). Antagonism of chemokine receptor CXCR3 inhibits osteosarcoma metastasis to lungs. Int J Cancer.

[21] Nakanishi T, Imaizumi K, Hasegawa Y, Kawabe T, Hashimoto N, Okamoto M, Shimokata K (2006). Expression of macrophage-derived chemokine (MDC)/CCL22 in human lung cancer. Cancer Immunol Immunother.

[22] Gyorffy B, Lanczky A, Eklund AC, Denkert C, Budczies J, Li Q, Szallasi Z (2010). An online survival analysis tool to rapidly assess the effect of 22, 277 genes on breast cancer prognosis using microarray data of 1,809 patients. Breast Cancer Res Treat.

[23] Buffa FM, Camps C, Winchester L, Snell CE, Gee HE, Sheldon H, Taylor M, Harris AL, Ragoussis J (2011). micro RNA-associated progression pathways and potential therapeutic targets identified by integrated mRNA and microRNA expression profiling in breast cancer. Cancer Res.

[24] Pang Y, Gara SK, Achyut BR, Li Z, Yan HH, Day CP, Weiss JM, Trinchieri G, Morris JC, Yang L (2013). Transforming growth factor beta signaling in myeloid cells is required for tumor metastasis. Cancer Discov.

[25] Ma B, Khazali A, Wells A (2015). CXCR3 in carcinoma progression. Histol Histopathol.

[26] Lasagni L, Francalanci M, Annunziato F, Lazzeri E, Giannini S, Cosmi L, Sagrinati C, Mazzinghi B, Orlando C, Maggi E, Marra F, Romagnani S, Serio M (2003). An alternatively spliced variant of CXCR3 mediates the inhibition of endothelial cell growth induced by IP-10, Mig, and I-TAC, and acts as functional receptor for platelet factor 4. J Exp Med.

[27] Singh AK, Arya RK, Trivedi AK, Sanyal S, Baral R, Dormond O, Briscoe DM, Datta D (2013). Chemokine receptor trio: CXCR3, CXCR4 and CXCR7 crosstalk via CXCL11 and CXCL12. Cytokine Growth Factor Rev.

[28] Li Y, Reader JC, Ma X, Kundu N, Kochel T, Fulton AM (2015). Divergent roles of CXCR3 isoforms in promoting cancer stem-like cell survival and metastasis. Breast Cancer Res Treat.

[29] Chaturvedi P, Gilkes DM, Wong CC, Luo W, Zhang H, Wei H, Takano N, Schito L, Levchenko A, Semenza GL (2013). Hypoxia-inducible factor-dependent breast cancer-mesenchymal stem cell bidirectional signaling promotes metastasis. J Clin Invest.

[30] Chaturvedi P, Gilkes DM, Takano N, Semenza GL (2014). Hypoxia-inducible factor-dependent signaling between triple-negative breast cancer cells and mesenchymal stem cells promotes macrophage recruitment. Proc Natl Acad Sci U S A.

[31] Lee JH, Kim HN, Kim KO, Jin WJ, Lee S, Kim HH, Ha H, Lee ZH (2012). CXCL10 promotes osteolytic bone metastasis by enhancing cancer outgrowth and osteoclastogenesis. Cancer Res.

[32] Hancock WW, Lu B, Gao W, Csizmadia V, Faia K, King JA, Smiley ST, Ling M, Gerard NP, Gerard C (2000). Requirement of the chemokine receptor CXCR3 for acute allograft rejection. J Exp Med.

[33] Pan J, Burdick MD, Belperio JA, Xue YY, Gerard C, Sharma S, Dubinett SM, Strieter RM (2006). CXCR3/CXCR3 ligand biological axis impairs RENCA tumor growth by a mechanism of immunoangiostasis. J Immunol.

[34] Mueller A, Meiser A, McDonagh EM, Fox JM, Petit SJ, Xanthou G, Williams TJ, Pease JE (2008). CXCL4-induced migration of activated T lymphocytes is mediated by the chemokine receptor CXCR3. J Leukoc Biol.

[35] Cekic C, Sag D, Li Y, Theodorescu D, Strieter RM, Linden J (2012). Adenosine A2B receptor blockade slows growth of bladder and breast tumors. J Immunol.

[36] Zumwalt TJ, Arnold M, Goel A, Boland CR (2015). Active secretion of CXCL10 and CCL5 from colorectal cancer microenvironments associates with GranzymeB + CD8 + T-cell infiltration. Oncotarget.

[37] Struyf S, Salogni L, Burdick MD, Vandercappellen J, Gouwy M, Noppen S, Proost P, Opdenakker G, Parmentier M, Gerard C, Sozzani S, Strieter RM, Van Damme J (2011). Angiostatic and chemotactic activities of the CXC chemokine CXCL4L1 (platelet factor-4 variant) are mediated by CXCR3. Blood.

[38] Wang Z, Huang H (2013). Platelet factor-4 (CXCL4/PF-4): an angiostatic chemokine for cancer therapy. Cancer Lett.

[39] Lu B, Humbles A, Bota D, Gerard C, Moser B, Soler D, Luster AD, Gerard NP (1999). Structure and function of the murine chemokine receptor CXCR3. Eur J Immunol.

[40] Ljung BM, Mayall B, Lottich C, Boyer C, Sylvester SS, Leight GS, Siegler HF, Smith HS (1989). Cell dissociation techniques in human breast cancer--variations in tumor cell viability and DNA ploidy. Breast Cancer Res Treat.

[41] Jessen KA, Liu SY, Tepper CG, Karrim J, McGoldrick ET, Rosner A, Munn RJ, Young LJ, Borowsky AD, Cardiff RD, Gregg JP (2004). Molecular analysis of metastasis in a polyomavirus middle T mouse model: the role of osteopontin. Breast Cancer Res.

[42] Le MT, Hamar P, Guo C, Basar E, Perdigao-Henriques R, Balaj L, Lieberman J (2014). miR-200-containing extracellular vesicles promote breast cancer cell metastasis. J Clin Invest.

