# Supplementary material for: CXCR3 as a molecular target in breast cancer metastasis: inhibition of tumor cell migration and promotion of host anti-tumor immunity
Source: Oncotarget. 2015 Oct 15;6(41):43408–19. doi: 10.18632/oncotarget.6125 (PMC4791240; doi:10.18632/oncotarget.6125)
Supplement: Supplementary file 1 [file oncotarget-06-43408-s001.pdf]

CXCR3 as a molecular target in breast cancer metastasis: inhibition of tumor cell migration and promotion of host anti-tumor immunity

Supplementary Materials

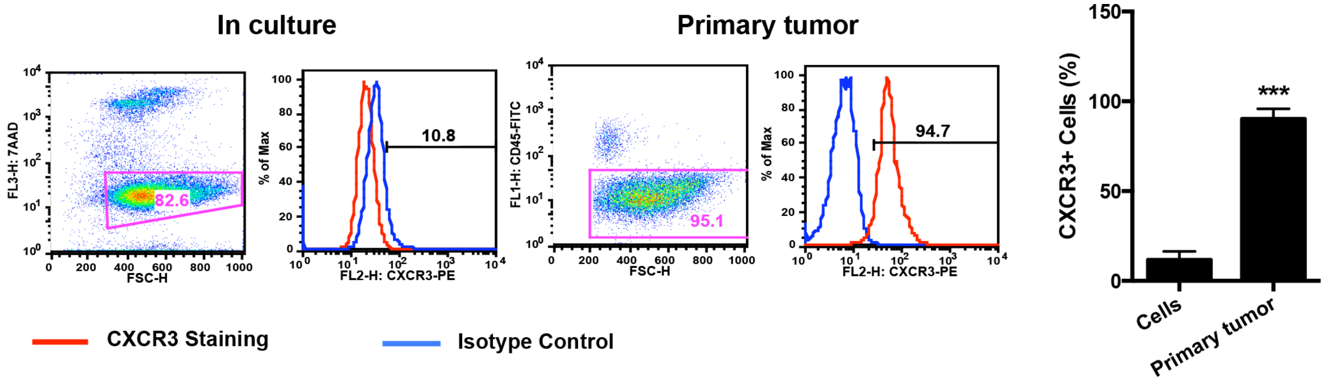

Supplementary Figure S1: Tumor cells at the primary tumor express higher CXCR3 than the ones cultured *in vitro*. Flow cytometry analysis of CXCR3 expression in single cell suspension from B16F10 primary tumor tissues compared B16F10 cells in culture. Single cell suspension was made using non enzymatic approach. The tumor cells were gated on 7AAD negative (exclusion of dead cells), CD45 negative (exclusion of immune cells), and large cells with high SSC and FSC scatter (tumor cells). The quantitative data is on the right. ***$P < 0.001$

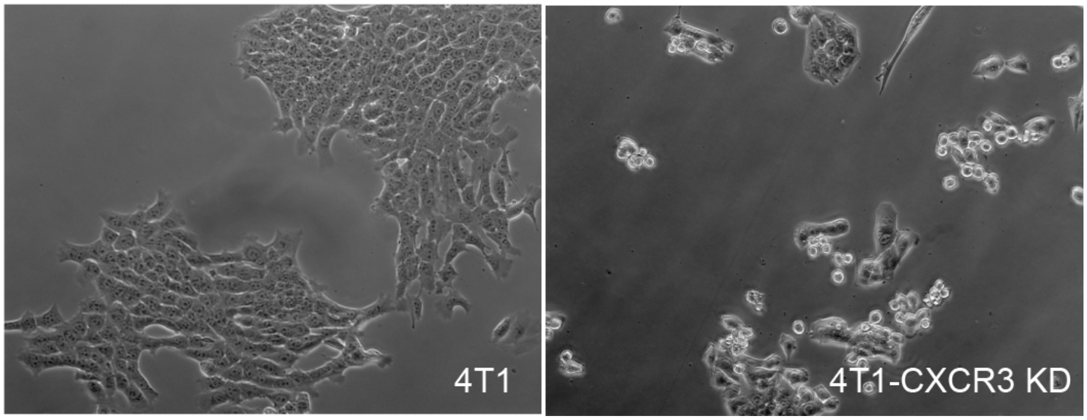

Supplementary Figure S2 : CXCR3 KD altered the morphology of 4T1 cells. Representative microscopy pictures showing the morphology of 4T1 tumor cells with or without CXCR3 KD.

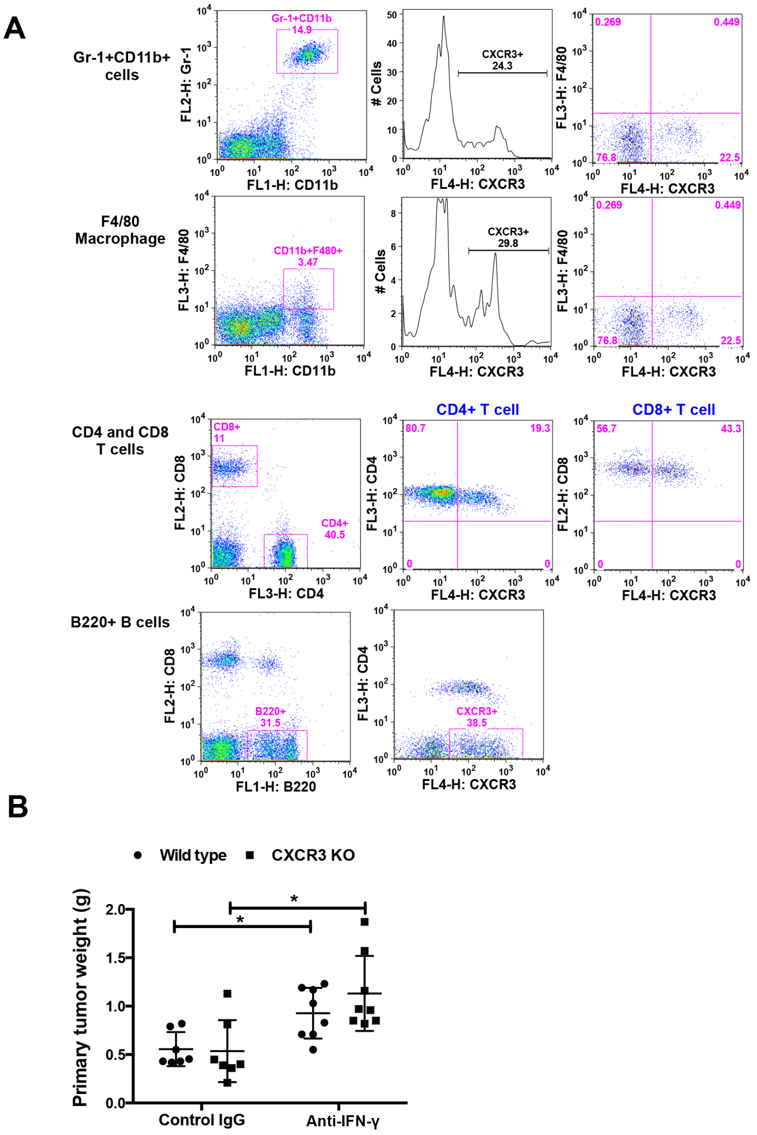

Supplementary Figure S3: Host CXCR3 promotes lung metastasis through suppression of host anti-tumor immunity. A. Flow cytometry analysis of CXCR3 expression in myeloid cells, T cells, and B cells. B. The tumor weight of wild type and CXCR3 KD mice bearing 4T1 tumors, with or without IFN-γ neutralization (figure 5D). 7–8 mice per group. *$P < 0.05$.

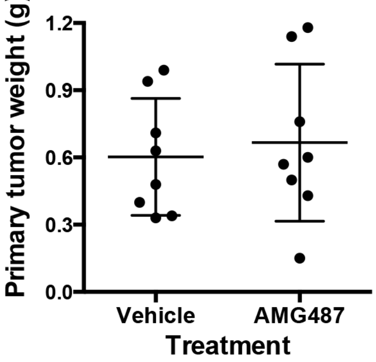

Supplementary Figure S4 : CXCR3 inhibitor AMG487 did not have an effect on primary tumor growth. Primary tumor sizes of 4T1 tumor-bearing mice treated with vehicle or AMG487. 11-12 mice per group.
